# Supplementary material for: Rapid learning with phase-change memory-based in-memory computing through learning-to-learn
Source: Nat Commun. 2025 Feb 1;16:1243. doi: 10.1038/s41467-025-56345-4 (PMC11787340; doi:10.1038/s41467-025-56345-4)
Supplement: Supplementary file 1 — Supplementary Information [file 41467_2025_56345_MOESM1_ESM.pdf]

Supplementary Information

Rapid learning with phase-change memory-based in-memory computing through learning-to-learn

Thomas Ortner[1†], Horst Petschenig[2†], Athanasios Vasilopoulos[1], Roland Renner[2], Špela Brglez[2], Thomas Limbacher[2], Enrique Piñero[3], Alejandro Linares-Barranco[3], Angeliki Pantazi[1], Robert Legenstein[2*]

[1]IBM Research Europe - Zurich, Säumerstrasse 4, Rüschlikon, 8803, Switzerland.
[2]Institute of Machine Learning and Neural Computation, Graz University of Technology, Inffeldgasse 16b, Graz, 8010, Austria.
[3]Robotics and Tech. of Computers. SCORE Lab. EPS-ETSII, Universidad de Sevilla, Av. Reina Mercedes, Seville, 41012, Spain.

*Corresponding author(s). E-mail(s): robert.legenstein@igi.tugraz.at;
†These authors contributed equally to this work.

Supplementary Figure 1

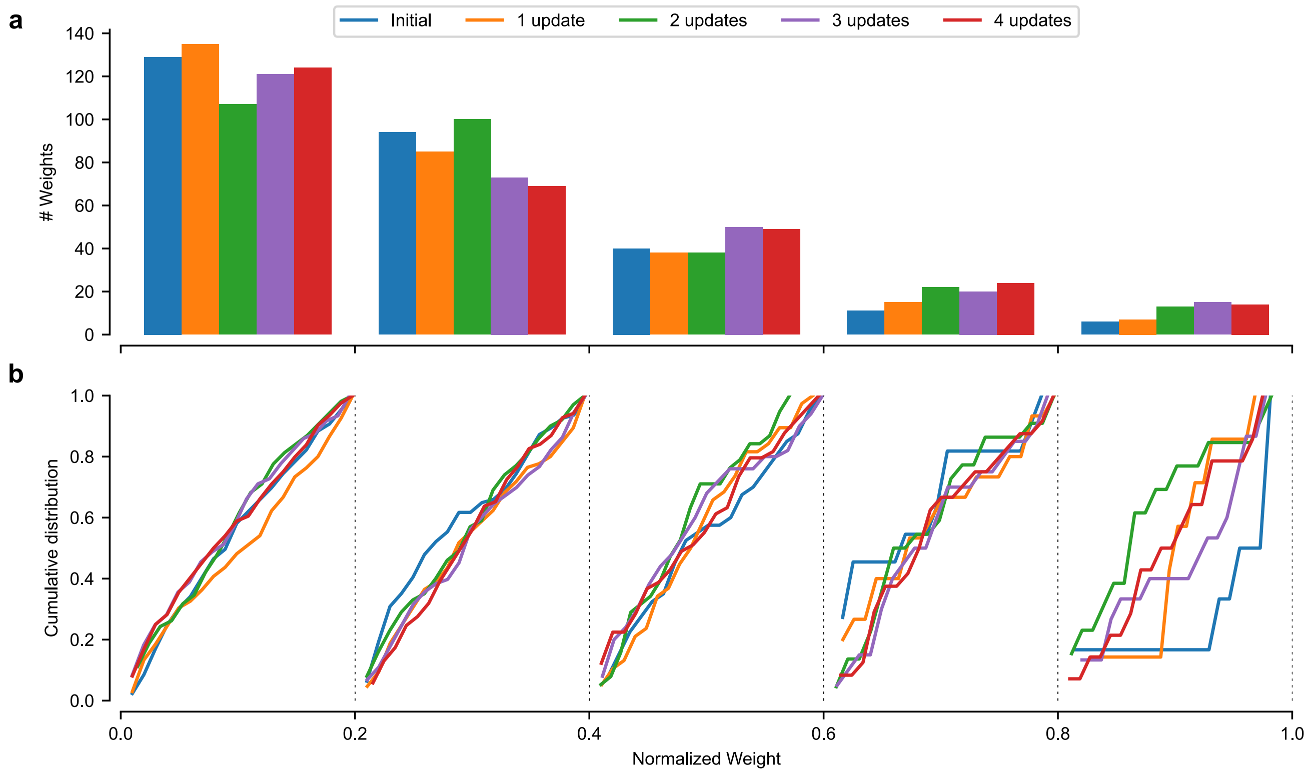

Fig. 1 Detailed analysis of dense layer.a Histogram and evolution of the normalized weights of the dense layer. The normalized weights are clustered into five bins, e.g., from 0. to 0.2, from 0.2 to 0.4, etc. The individual bars within the bins show the weights before any update (blue bar), after the first update (orange bar), after the second update (green bar), after the third update (purple bar) and after the fourth update (red bar). **b** Cumulative distribution of the weights within the individual bins. While the weight values within the first few bins remain rather unchanged, the weight distribution within the last two bins changes significantly over the course of the updates.

Supplementary Figure 2

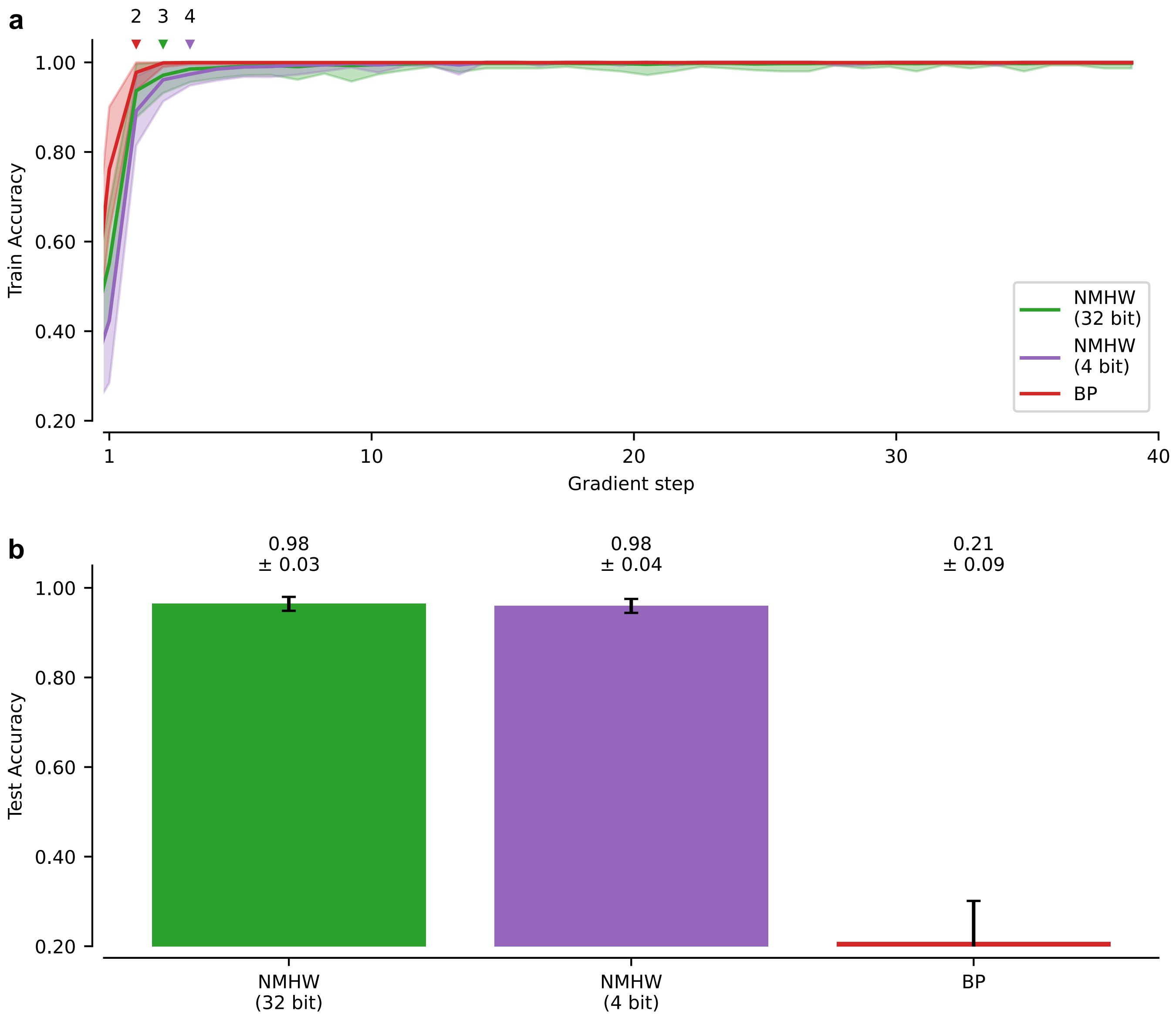

Fig. 2 Comparison of task-adaption with a MAML-meta-trained network and a network that is trained from scratch with BP. a Train accuracy during task-adaptation on the 5-way 5-shot Omniglot classification experiment as outlined in Section 2.2 of the main manuscript. The plot shows the evolution of the train accuracy for our model on the NMHW with 32 bit (green curve), on the NMHW with 4 bit (purple curve) and for the from-scratch trained network with BP (red curve). The triangular markers on top of the panel indicate the gradient step at which the train accuracy exceeds 97%. **b** Test accuracy for 25 unseen test images (from the classes on which the networks were trained) of the models from **a** after training. While our models on the NMHW are able to classify the test images almost perfectly, test accuracy of around 98%, the network trained with BP struggles to generalize and only achieves a test accuracy of around 20%.

Supplementary Figure 3

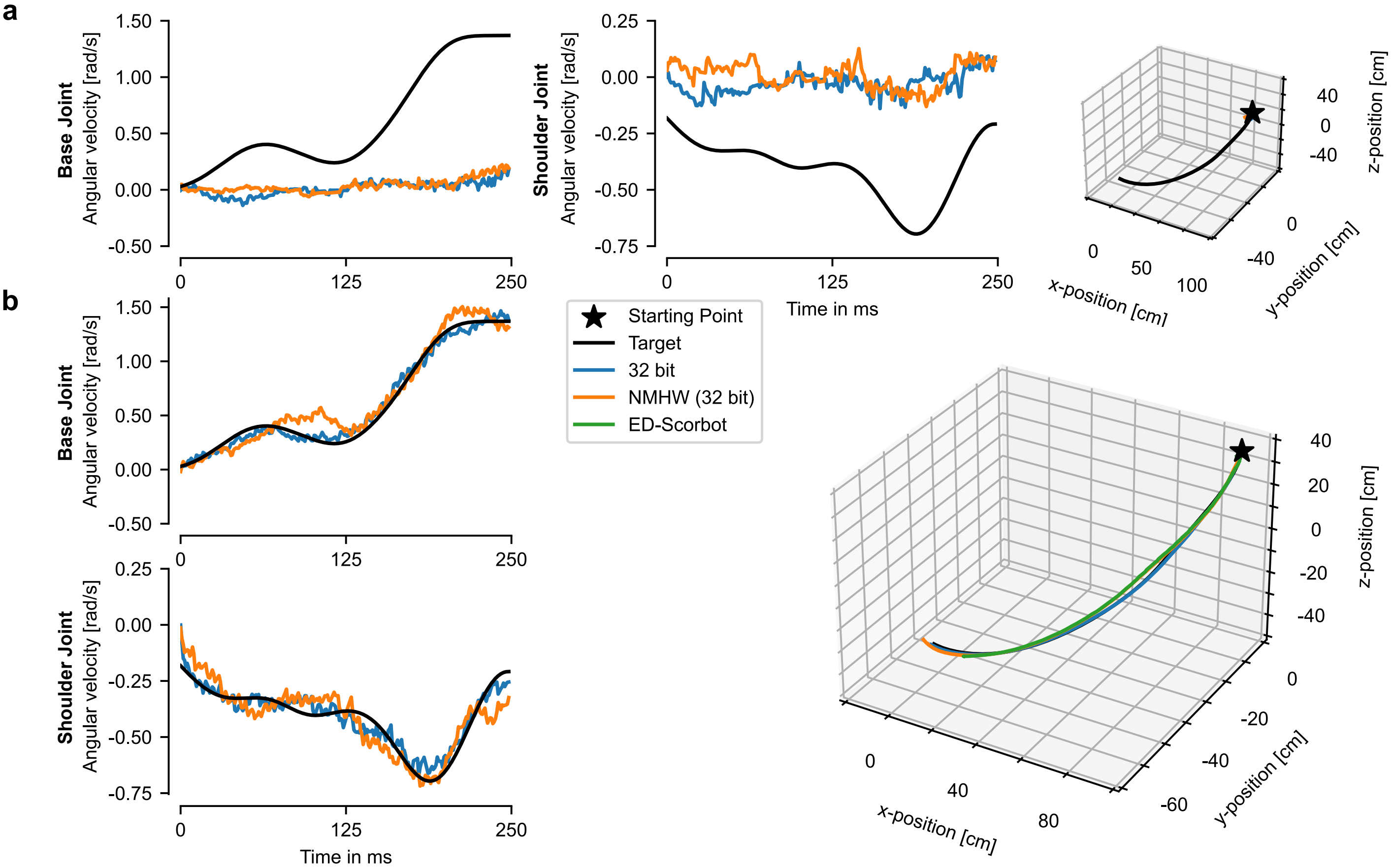

Fig. 3 Evaluation of a second trajectory for the robotic task described in Section 2.3 of the main manuscript. **a** Angular velocities and trajectories in the Euclidean space of the meta-trained network in software (blue) with NMHW (orange) before the inner loop update. **b** Angular velocities and trajectories in the Euclidean space of the networks after one-shot learning. The green trajectory shows the trajectory of the ED-Scorbot robot.

Supplementary Figure 4

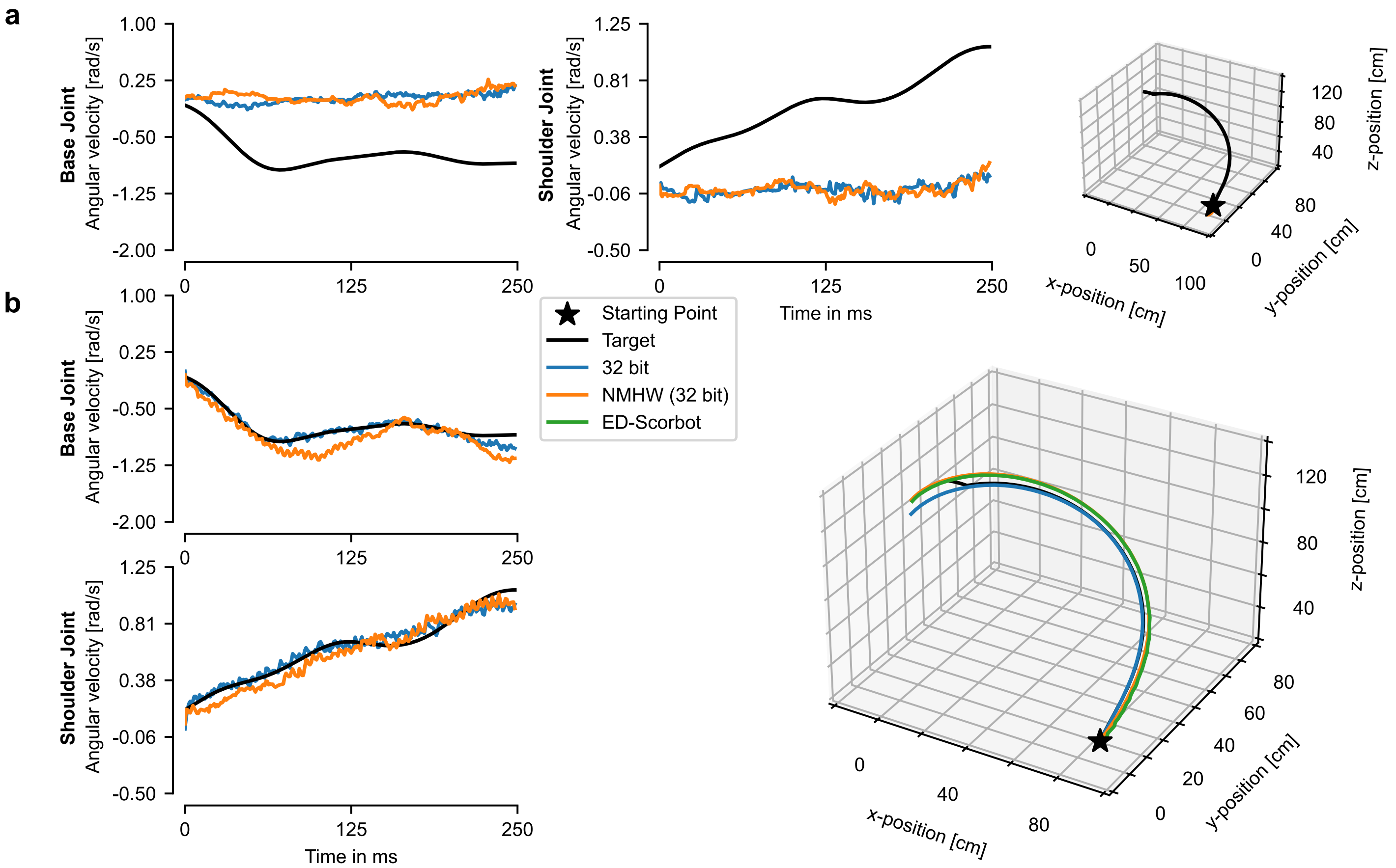

Fig. 4 Evaluation of a third trajectory for the robotic task described in Section 2.3 of the main manuscript. **a** Angular velocities and trajectories in the Euclidean space of the meta-trained network in software (blue) with NMHW (orange) before the inner loop update. **b** Angular velocities and trajectories in the Euclidean space of the networks after one-shot learning. The green trajectory shows the trajectory of the ED-Scorbot robot.

Supplementary Figure 5

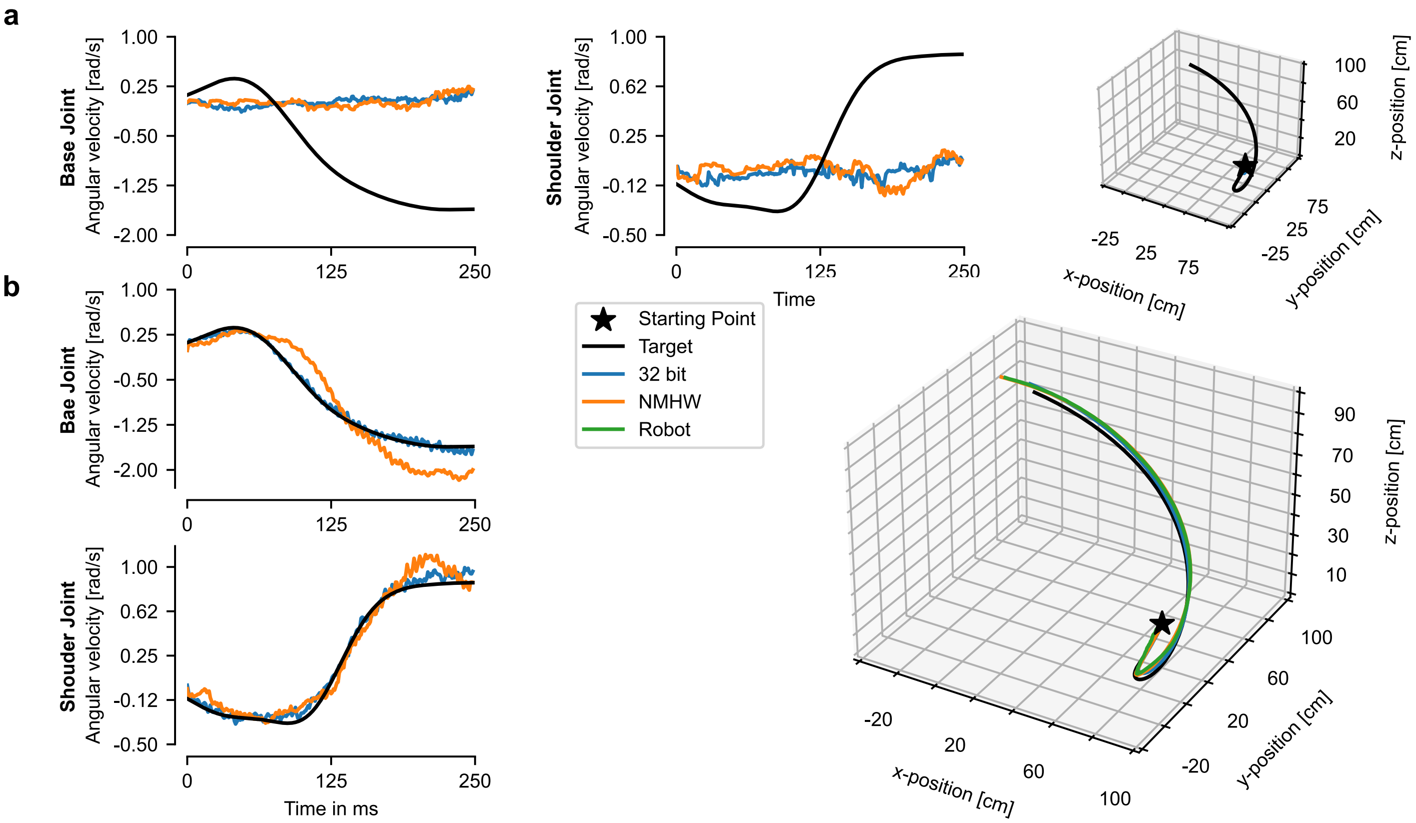

Fig. 5 Evaluation of a fourth trajectory for the robotic task described in Section 2.3 of the main manuscript. **a** Angular velocities and trajectories in the Euclidean space of the meta-trained network in software (blue) with NMHW (orange) before the inner loop update. **b** Angular velocities and trajectories in the Euclidean space of the networks after one-shot learning. The green trajectory shows the trajectory of the ED-Scorbot robot.

Supplementary Figure 6

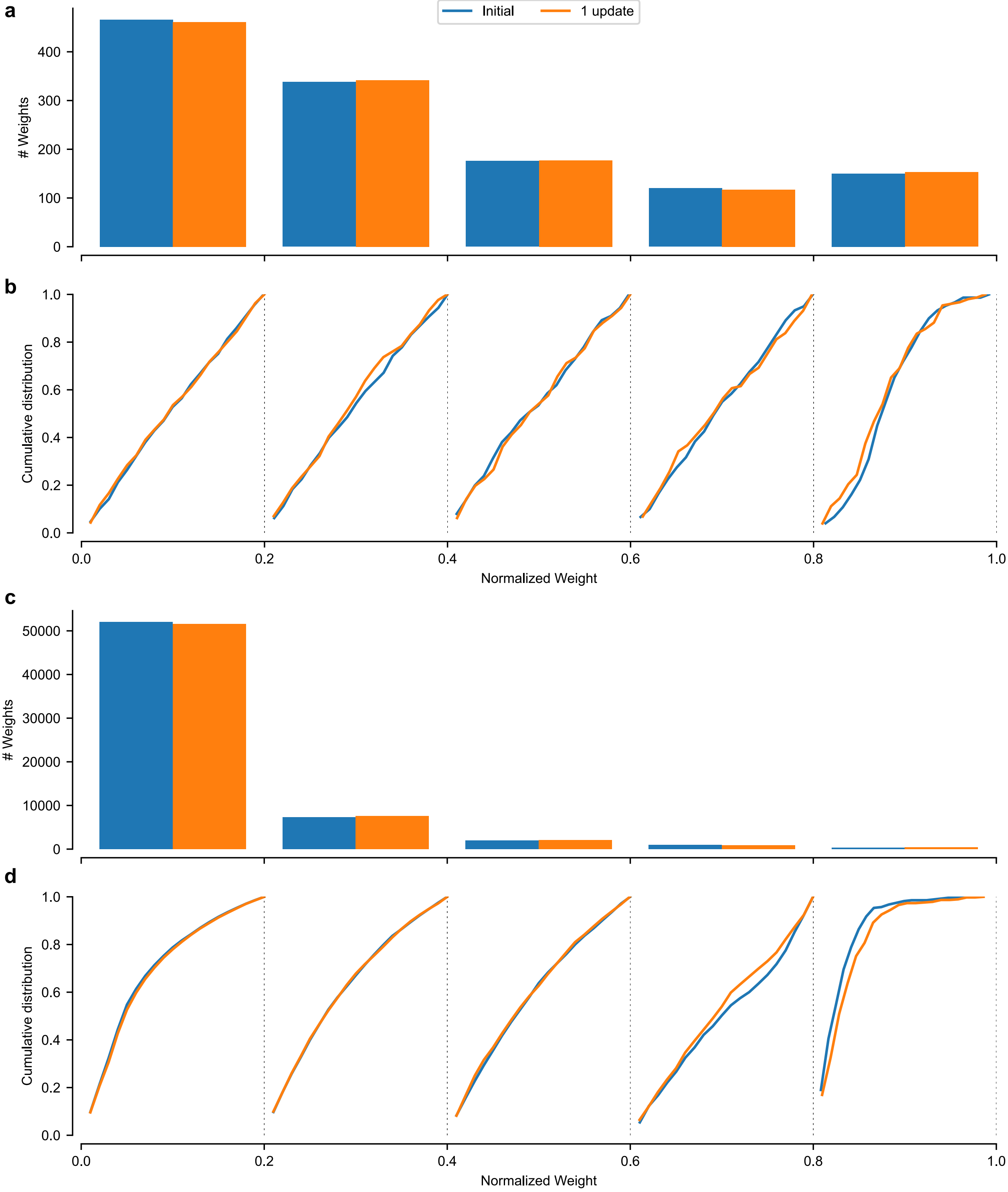

Fig. 6 Detailed analysis trainee from Section 2.3 of the main manuscript.a Histogram and evolution of the normalized weights of the input weights of the trainee $\boldsymbol{\theta}^{\text{in}}$. The normalized weights are clustered into five bins, e.g., from 0. to 0.2, from 0.2 to 0.4, etc. The individual bars within the bins show the weights before any update (blue bar) and after the single-shot update (orange bar). **b** Cumulative distribution of the weights within the individual bins for the input weights. While the weight values within the first few bars remain unchanged, the weight distribution within the last two bins tends to change more over the course of the updates. **c** Histogram and evolution of the normalized weights of the recurrent weights of the trainee $\boldsymbol{\theta}^{\text{rec}}$. **d** Cumulative distribution of the weights within the individual bins for the recurrent weights. The weight distributions within the first three bins tends to change little over the course of the updates, and the main changes of the weights appear in the last two bins (the larger weights).

Literature comparison

Related work	Network	Training algorithm<br>Outer loop	 Inner loop	Tasks	Datasets	NMHW
Bohnstingl [1]	SNN	Cross-Entropy<br>Evolution Strategies<br>Simulated Annealing<br>Gradient Descent	Q-Learning<br>TD(1)-Learning<br>TD(λ)-Learning	Reward Max.	Bandit Tasks<br>MDP	HICANN-DLSv2<br>(no PCM)
Zhang [2]	CNN	MAML	Gradient Descent	Image proc.	Omniglot<br>MiniImageNet	Simulated PCM
Wu [3]	SNN<br>Spk. CNN	BPTT	Hebbian	Image proc.	(F,S,N)-MNIST<br>CIFAR10<br>CIFAR10-DVS<br>DVS-Gesture<br>Omniglot	Tianjic<br>(no PCM)
Yu [4]	CNN	MemMAML	Gradient Descent	Image proc.	Omniglot	Simulated<br>Memristive devices
**Ours**	**CNN**<br>**SNN**	**MAML**<br>**Natural e-prop**	**Gradient Descent**<br>**e-prop**	**Image proc.**<br>**Robotic task**	**Omniglot**<br>**CIFAR100-FS**<br>**Rnd. Trajectories**	**Physical PCM**

Table 1 Comparison of related works leveraging learning-to-learn and neuromorphic hardware.

References

[1] Bohnstingl, T., Scherr, F., Pehle, C., Meier, K., Maass, W.: Neuromorphic hardware learns to learn. Frontiers in Neuroscience **13**, 451952 (2019)

[2] Zhang, W., Wang, Y., Ji, X., Wu, Y., Zhao, R.: Roa: a rapid learning scheme for in-situ memristor networks. Frontiers in Artificial Intelligence **4**, 692065 (2021)

[3] Wu, Y., Zhao, R., Zhu, J., Chen, F., Xu, M., Li, G., Song, S., Deng, L., Wang, G., Zheng, H., *et al.*: Brain-inspired global-local learning incorporated with neuromorphic computing. Nature Communications **13**(1), 65 (2022)

[4] Yu, Z., Leroux, N., Neftci, E.: Training-to-Learn with Memristive Devices. In: 2022 International Electron Devices Meeting (IEDM), pp. 21–112114 (2022)
