# Supplementary material for: Rapid learning with phase-change memory-based in-memory computing through learning-to-learn
Source: Nat Commun. 2025 Feb 1;16:1243. doi: 10.1038/s41467-025-56345-4 (PMC11787340; doi:10.1038/s41467-025-56345-4)
Supplement: Supplementary file 2 — Description of Additional Supplementary Information [file 41467_2025_56345_MOESM2_ESM.docx]

**Description of Additional Supplementary Files**

File Name: Supplementary Movie 1

Description: Demonstration of rapid online learning of motor commands with neuromorphic hardware. Left: Trajectory in the Euclidean space. Target trajectory shown in black and ED-Scorbot in green. Right: Movement of the real ED-Scorbot.
